# Context Is Key: Delineating the Unique Functions of IFNα and IFNβ in Disease

**DOI:** 10.3389/fimmu.2020.606874

**Published:** 2020-12-21

**Authors:** Lindsey E. Fox, Marissa C. Locke, Deborah J. Lenschow

**Affiliations:** ^1^ Department of Pathology and Immunology, Washington University School of Medicine, Saint Louis, MO, United States; ^2^ Department of Medicine, Washington University School of Medicine, Saint Louis, MO, United States

**Keywords:** type I interferons, infection, autoimmunity, cancer, IFNα subtypes, IFNβ

## Abstract

Type I interferons (IFNs) are critical effector cytokines of the immune system and were originally known for their important role in protecting against viral infections; however, they have more recently been shown to play protective or detrimental roles in many disease states. Type I IFNs consist of IFNα, IFNβ, IFNϵ, IFNκ, IFNω, and a few others, and they all signal through a shared receptor to exert a wide range of biological activities, including antiviral, antiproliferative, proapoptotic, and immunomodulatory effects. Though the individual type I IFN subtypes possess overlapping functions, there is growing appreciation that they also have unique properties. In this review, we summarize some of the mechanisms underlying differential expression of and signaling by type I IFNs, and we discuss examples of differential functions of IFNα and IFNβ in models of infectious disease, cancer, and autoimmunity.

## Introduction

Interferons (IFNs) are cytokines that were originally discovered and named for their ability to interfere with viral replication (1). IFNs are grouped into three classes according to the receptor that mediates their effects: type I IFNs (the focus of this review), type II IFN (IFNγ), and type III IFNs (IFNλs) (2, 3). Broadly speaking, each IFN class signals through receptor-associated Janus kinases (JAKs), which activate various Signal Transducer and Activator of Transcription (STAT)-signaling pathways. Type I IFNs signal through the heterodimeric IFN-α/β receptor 1 (IFNAR1) and IFNAR2, which are associated with the JAKs tyrosine kinase 2 (TYK2) and JAK1, respectively (4). Canonically, activation of TYK2 and JAK1 leads to the formation of the IFN-stimulated gene (ISG) factor 3 (ISGF3) complex, composed of STAT1, STAT2, and interferon regulatory factor 9 (IRF9). The ISGF3 complex then translocates to the nucleus to regulate the expression of hundreds of IFN-stimulated genes. Type I IFN signaling can activate other STAT complexes, often in a cell-type dependent manner. Additionally, alternative signaling cascades, including the mitogen-activated protein kinase p38 pathway and the phosphatidylinositol 3-kinase pathway, are also required for optimal generation of type I IFN responses (4).

Type I IFNs have broad, pleiotropic effects that include antiviral activity, antiproliferative effects, and immunomodulatory properties. There is growing evidence that the overall outcome of type I IFN responses can be beneficial or detrimental for the host depending on the timing, magnitude, and source of IFN production, as well as the specific biological context (5). Moreover, despite signaling through a shared receptor, type I IFN subtypes possess important functional differences, both *in vitro* and *in vivo*. The purpose of this review is to summarize the current understanding of differential type I IFN properties, focusing on the role of human and mouse IFNα and IFNβ in infectious disease, cancer, and autoimmunity. In particular, we seek to highlight the few examples that demonstrate or suggest differential activities for type I IFN subtypes *in vivo*.

## Type I IFNS: A Multigene Family

**Figure 1:**
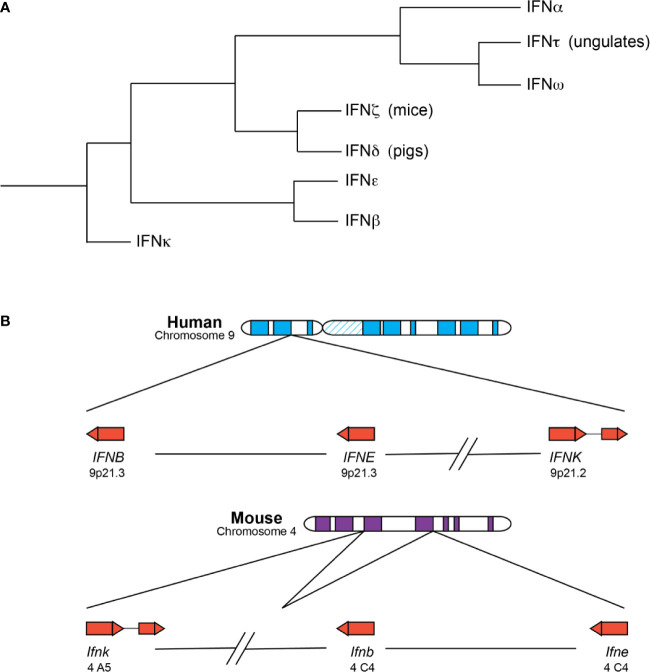
Type I IFNs are a closely related family of related cytokines. **(A)** Depicted is a summary of existing phylogenetic analyses of the type I IFNs. The branches are not drawn to scale. IFNκ, IFNβ, and IFNϵ are mostly present in placental mammals as single copies and the first subtypes to diverge from the other type I IFNs. IFNβ and IFNϵ are especially similar and can be found within the same clade in some analyses. IFNδ and IFNζ are the next subtypes to diverge and are only found in pigs and mice, respectively. IFNτ and IFNω are closely related, despite their differences in function and distribution—IFNτ is only expressed in placental tissues of ungulate species and involved in pregnancy, whereas IFNω is found in many species and possesses the more canonical antiviral and immunomodulatory functions. IFNω and IFNα loci are expanded to include many subtypes in a number of species. **(B)** The chromosomal locations of human (top) and murine (bottom) IFNκ, IFNβ, and IFNϵ genes are depicted. The arrow direction indicates on which strand the gene is encoded: a left-to-right arrow depicts the forward or positive strand and a right-to-left arrow indicates the reverse or negative strand. IFNκ is the only subtype to contain an intron and is situated further away from the other type I IFNs, though its positioning relative to the other IFNs is different in mice and humans. IFNβ and IFNϵ roughly form the boundaries of the type I IFN locus, with the other type I IFNs falling between the two genes.

Type I IFNs exist as a multigene family across many species (**Figure 1**) **(** 6). IFNαs, IFNβ, IFNϵ, IFNκ, and IFNω are found in many species, whereas IFNδ and IFNτ are only found in pigs and cattle (7). In humans (HuIFN), the type I IFN genes are located on chromosome 9 and encode 13 IFNα subtypes and single forms of IFNβ, IFNϵ, IFNκ, and IFNω (7). Type I IFNs in mice (MuIFN) are located on chromosome 4, and likewise, consist of multiple genes with some differences compared to human. MuIFNs include 14 IFNα subtypes, IFNβ, IFNϵ, IFNκ, an IFN-like cytokine IFNζ (also known as limitin), but lack a functional IFNω, which is present as a pseudogene (8).

Phylogenetic analyses reveal that the type I IFN subtypes form clades consistent with mammalian speciation (7, 9, 10). For the most part, placental mammals possess single copies of the genes encoding IFNκ, IFNβ, and IFNϵ, and these unduplicated subtypes represent the first major clade within mammalian IFNs (11). IFNκ is the first subtype to diverge within mammalian type I IFNs and forms an outgroup, possibly the result of a unique evolutionary route for IFNκ relative to IFNβ and IFNϵ (11). IFNκ is additionally distinctive as the only mammalian type I IFN that contains an intron, and for many species, the gene encoding IFNκ is situated further away from the IFN locus (7, 9, 11). Depending on the analysis, IFNβ or IFNϵ is the next subtype to diverge from mammalian type I IFNs, and in some analyses IFNβ and IFNϵ fall within the same clade, suggesting that these subtypes might be more closely related to each other than the other type I IFN subtypes (7, 9, 11, 12). The genes encoding IFNβ and IFNϵ are situated at the “beginning” and “end” of the type I IFN locus across many species, which is relatively conserved across mammalian species. IFNδ and IFNζ (limitin) are the next type I IFNs to diverge within mammalian IFNs and are only found in pigs and mice, respectively (7). However, recent identification of a putative HuIFNδ gene calls this into question (11).

The last subtypes to diverge are the IFNαs, IFNωs, and IFNτs. These subtypes are thought to be exclusively found in placental mammals and are usually situated between the IFNϵ and IFNβ genes within the type I IFN locus. IFNω and IFNτ are closely related, even though they possess different functions (7, 11). IFNτ is only found in placental tissues of ungulate species, is involved in pregnancy, and may have arisen from an IFNω subtype (10, 13). In contrast, IFNω is an antiviral and immunomodulatory molecule, like IFNα, and functional copies have been identified in humans and other animal groups including felines, pigs, cattle, serotine bats, and others but are not present in canines or mice (14). Notably, humans have only one IFNω, but there is evidence that IFNω is still expanding and diversifying in many species, including bats and pigs (15–17). Lastly, the genes encoding IFNα are found in all placental mammals and form species-specific clades, with some exceptions for closely related organisms (e.g. chimpanzees, humans, and gorillas); a combination of gene duplication and gene conversion events likely gave rise to the expanded IFNα genes present in many mammals (6). Of note, a recent study found that for some IFNα subtypes, such as HuIFNα6, α8, α13, and α14, amino acid-altering variation was more constrained in the human population, suggesting that they might perform non-redundant functions in host responses (18).

As sequenced genomes of other species become available, the phylogenetic clustering of some type I IFNs may change. However, the key point is that the multigene nature of type I IFNs is conserved across many species. Both IFNα and IFNω subtypes expanded independently and multiple times, suggesting that it is advantageous for the host to possess a large repertoire of at least several type I IFN subtypes. Unfortunately, the fact that type I IFNs expanded multiple times complicates directly applying results of IFN studies from animal models to clinical settings, and caution is warranted in drawing conclusions about specific human IFNα subtypes from studies of murine IFNα subtypes.

## Molecular Mechanisms Underlying Distinct Functions of Type I IFNs

Though type I IFNs possess many overlapping functions, it is now appreciated that the individual subtypes have different potencies of their shared functions and some unique functions *in vitro*. An important early example demonstrating this was the finding that HuIFNβ was 100-fold more potent than HuIFNα2 in inhibiting osteoclastogenesis through its ability to preferentially induce the chemokine CXCL11 (19). Since this observation, it is now appreciated that the pleiotropic activities ascribed to different type I IFN subtypes are the product of distinct patterns and kinetics of expression, as well as signaling differences that arise from differential binding affinities and susceptibility to negative feedback loops (20, 21). The ability of the type I IFN receptor to have fine-tuned responses to many ligands is likely advantageous considering the array of pathogens that have co-evolved alongside humans, mice, and other animals.

### Differential Dependence on IRF3 and IRF7 for Transcription

Before examining the signaling and functional properties of IFN subtypes, it should be noted that type I IFNs are differentially induced downstream of pattern recognition receptor (PRR) signaling, except for IFNϵ, which is hormonally regulated (see below). PRR signaling converges on the phosphorylation and activation of the transcription factors IRF3 and IRF7, though other IRFs can be involved in IFN-dependent antiviral responses (22, 23). For most cell types IRF3 is constitutively expressed, whereas IRF7 is induced downstream of type I IFN signaling to then amplify and diversify the type I IFN response (22). The exception to this rule is plasmacytoid dendritic cells (pDCs), which constitutively express IRF7 and are thus poised to rapidly secrete large amounts of type I IFN (24). The promoters of specific type I IFN genes differ in their requirement of IRF3 or IRF7 binding for maximal transcription. Thus, the temporal regulation of IRFs dictates the expression of IFN subtypes.

Early in a response, IRF3 activation first induces transcription of MuIFNβ and MuIFNα4 *via* unique IRF3 binding sites within their promoters (25–31). For the most part, the other MuIFNα subtypes require both IRF3 and IRF7 for maximal transcription, and so they depend on type I IFN-mediated upregulation of IRF7 (32–34). Similar to mice, IRF3 also initiates human type I IFN responses by upregulating transcription of HuIFNβ and HuIFNα1, while the other HuIFNA genes require both IRF3 and IRF7 (35, 36). Altogether, these findings demonstrate that for most cell types, activation of constitutive IRF3 by PRR signaling initiates a first wave of HuIFNβ and HuIFNα1 (or MuIFNβ and MuIFNα4 for mice). Subsequently, a second, amplified wave of diverse IFNα subtypes follows that is IRF7-dependent. As the ratio of IRF3 to IRF7 or other IRFs changes over time, the repertoire of IFN subtypes expressed changes as well.

There are several intriguing deviations from this paradigm. First, the IFNβ promoter has additional response elements that make it responsive to NF-κB signaling through activating transcription factor 2 (ATF-2) and c-Jun, which allows other signaling pathways to augment IFNβ production (29, 37, 38). This unique promoter feature also permits IRF3-independent basal expression of low amounts of IFNβ in the absence of infection, which can have significant impact on mounting successful innate immune responses against a variety of infections (39–47). IFNκ may have somewhat restricted expression, as it was named for its high expression in keratinocytes; however, other cell types, including immune cells and lung epithelial cells, can upregulate IFNκ expression (48–50). Further characterization is needed to determine which cells are capable of expressing IFNκ in different contexts. Lastly, IFNϵ is the most notable exception to the IRF-mediated IFN induction paradigm, as it is not regulated at all by PRR signaling and IRF3/7. Instead, it is constitutively expressed in the epithelium of reproductive organs and hormonally regulated, and this is reflected in its unique promoter (51–53).

### Differential Binding Affinity Determines Signaling and Function

All type I IFNs bind to and signal through the heterodimeric receptor IFNAR1 and IFNAR2 to activate canonical JAK/STAT signaling pathways (4). A unique feature of type I IFN signaling is that the signaling outcome can vary depending on the cell type, specific ligand, and concentration of the type I IFN subtype. The molecular mechanisms that underlie the plasticity of type I IFN signaling have been extensively reviewed elsewhere, so only key features will be outlined in this review (20, 54, 55).

In general, IFNAR2 is the primary ligand binding receptor subunit and binds type I IFNs with high affinity (typically nanomolar affinity); IFNAR1 is subsequently recruited to the receptor-ligand complex and binds with relatively lower affinity (approximately micromolar affinity) (54). HuIFNβ has the highest natural binding affinity to the type I IFN receptors with picomolar affinity for IFNAR2 and nanomolar affinity for IFNAR1, whereas HuIFNα2 possesses nanomolar affinity for IFNAR2 and micromolar affinity for IFNAR1 (56–58). This higher affinity interaction may enable IFNβ to uniquely signal through IFNAR1 in an IFNAR2-independent manner, but further work is needed to corroborate this finding and to determine if other receptors are involved in this phenomenon (59, 60). Engineered IFNα2 and IFNω mutants that mimic the range of affinities for the receptor complex have demonstrated that type I IFN signaling outcomes can be directly linked to IFN affinity to the receptor complex. Hence, type I IFN mutants that acquire IFNβ-like affinity acquire IFNβ-like potency (61, 62).

In line with these findings for IFNα, IFNβ, and IFNω, recent work showed that HuIFNϵ and HuIFNκ bound IFNAR2 with particularly weak affinity and demonstrated approximately 1000-fold decreased potency in ISGF3-mediated gene expression compared to HuIFNα2, whereas their affinity for IFNAR1 was comparable to other type I IFN subtypes (63). HuIFNϵ and HuIFNκ also bound the poxvirus antagonist B18R with weaker affinity relative to the other IFN subtypes, perhaps suggesting a fitness advantage for the host to have some weaker binding IFN subtypes in order to avoid virus inhibition (63). In influenza A virus (IAV) infection, HuIFNκ, but not IFNα or IFNβ, relied on chromodomain helicase DNA binding protein 6 (CHD6) to efficiently suppress viral replication (50). Moreover, induction of CHD6 was not dependent on STAT1, but rather, IFNκ signaled through the mitogen-activated protein kinase (MAPK) p38 and the transcription factor c-Fos to mediate its antiviral effects. Altogether, these findings suggest that in addition to having unique expression patterns, IFNϵ and IFNκ may possess additional biochemical and signaling features that grant unique properties *in vivo*.

### Differential Sensitivity to Feedback Loops

The affinity of individual subtypes, as outlined above, is a key component in determining the signaling outcome from IFNAR1/2 engagement, but negative feedback loops are an additional level of regulation and fine-tuning. IFNAR1/2 surface abundance is typically quite low, and modulating the surface receptor expression is one means of regulating type I IFN signaling after type I IFN induction (64). Manipulation of a cell line’s IFNAR expression demonstrated that the antiproliferative and proapoptotic activities induced by HuIFNβ are less sensitive to decreased receptor levels than those induced by HuIFNα2 (65, 66). The physiological relevance of receptor expression influencing type I IFN signaling is demonstrated in the number of IFN-dependent mechanisms that downregulate IFNAR1 and IFNAR2 levels. We will outline a few examples.

First, protein kinase D2 (PKD2) is a negative regulator activated downstream of IFN signaling. It phosphorylates IFNAR1, enabling interaction with a ubiquitin E3 ligase, and subsequent ubiquitination leads to endocytosis of the IFN signaling complex (67, 68). Endosomes with short-lived receptor-ligand complexes formed by lower affinity IFNαs are more likely to be recycled to the cell surface; endosomes with longer-lived complexes formed by higher affinity IFNβ ultimately fuse with the lysosome, but signaling can continue to take place as trafficking progresses through the endosomal compartment (69–72). Second, Suppressor of Cytokine Signaling 1 (SOCS1) can directly dampen the type I IFN response by interacting with TYK2 to disrupt TYK2-STAT signaling, but it also decreases surface levels of IFNAR1, which requires TYK2 for stability at the cell surface (73). Lastly, ubiquitin-specific peptidase 18 (USP18) can bind the cytoplasmic domain of IFNAR2 and interfere with IFNAR1 recruitment and ternary receptor complex formation without decreasing surface IFNAR2 levels (74, 75). The USP18-IFNAR2 interaction makes it so that only higher affinity ligands such as IFNβ are able to recruit IFNAR1 into the receptor complex, making the cell less responsive to weaker affinity type I IFNs (76, 77).

### Key Principles for Differential Activities

Altogether, differential expression, binding affinity to the receptor, and downstream feedback loops enable IFNAR1/2 to have graded responses to multiple ligands. Redundancy and pleiotropy are key features of type I IFN responses. Essentially, any type I IFN subtype can induce robust (or redundant) properties, such as antiviral activity, even at low surface receptor density. In contrast, tunable (or pleiotropic) functions, like antiproliferative activity, are more heavily influenced by affinity of the ligand, receptor density, and intracellular negative regulators, and so higher affinity ligands, like IFNβ, tend to be more potent (21). However, as noted above, some type I IFN subtypes may be able to signal through alternative pathways, in spite of or, more likely, because of possessing lower binding affinity. Understanding the molecular mechanisms underlying differential signaling by IFNs is an active area of research and how the differential activities of IFNα and IFNβ impact disease will be explored in the remaining sections.

## Infectious Diseases

Type I IFNs have been extensively studied in the context of infectious diseases, and this body of work includes most of the studies that have directly compared the functions of IFNα and IFNβ *in vivo*. In the following subsections we highlight key findings from animal models and human studies that have contributed to understanding the mechanisms of differential properties of IFNα and IFNβ in viral, bacterial, and parasitic infections.

### Viral Infections

The important role that viral infections have served in helping us understand type I IFN biology cannot be understated. Viral infections were key instruments in the discovery of the antiviral properties of type I IFNs (1). It is now widely appreciated that type I IFNs play a much larger role in coordinating protective immunity beyond directly eliciting an antiviral state, including their role in DC maturation, augmenting antibody production by B cells, and improving cytolytic T cell effector functions (5). Intriguingly, type I IFNs can also play a detrimental role in certain contexts, such as persistent viral infections. Given their key roles in disease outcome, viral systems also include some of the clearest examples of differential functions of IFNα and IFNβ *in vivo* (**Table 1**). The following viral models collectively highlight that differential functions of IFNαs and IFNβ can profoundly influence disease pathogenesis and that the mechanisms underlying differential functions vary depending on the biological context.

**Table 1:** Summary of IFNα and IFNβ functions in mouse models of viral infections.

Intervention	Clinical Outcome	Virological and Immune Characterization	Refs.
**Lymphocytic choriomeningitis virus Cl-13, i.v. (persistent infection)**			
*Ifnar1^-/-^* or αIFNAR1 mAb	Improved splenic architecture	V: ↑ viremia, ↑ tissue titers (early); ↓ viremia, ↓ tissues titers (late) I: ↓ IL10 (serum), ↓ PD-L1 expression (splenic cells), ↑ Ag^+^ CD4 T (spleen)	(78–80)
αIFNα mAb	ND	V: ND (early); no Δ viremia, ↑ splenic titer (late)	(80)
*Ifnb^-/-^* or αIFNβ mAb	Improved splenic architecture	V: no Δ (early); ↓ viremia, ↓ tissue titers (late) I: no Δ IL-10 (serum), no Δ PD-L1 expression (splenic cells), ↑ Ag^+^ CD4 T (spleen)	(80)
**Lymphocytic choriomeningitis virus Cl-13, i.v. (lethal infection)**			
NZB.*Ifnar1^-/-^* or αIFNAR1 mAb (NZB)	↓ vascular leakage, ↓ lethality (0%)	V: ↑ viremia that persists I: ↓ CTL activity, ↓ lung infiltrate, ↓ BALF cytokines	(81)
NZB.*Ifnb^-/-^*	No Δ lethality	ND	(81)
αIFNAR1 mAb (FVB/N or NZO)	↓ vascular leakage, ↓ lethality (0%)	V: ↑ viremia that persists I: ↑ platelet count	(82)
αIFNβ mAb (FVB/N)	No Δ lethality	ND	(82)
αIFNα mAb (FVB/N)	No Δ lethality	ND	(82)
αIFNα and αIFNβ mAbs co-treatment (FVB/N)	No Δ lethality	ND	(82)
**West Nile virus, s.c. (footpad)**			
*Ifnar1^-/-^*	↑ lethality (100%)	V: ↑ viremia, ↑ tissue titers	(83, 84)
*Irf7^-/-^*	↑ lethality (100%)	V: ↑ viremia, ↑ tissue titers I: ↓ serum IFNα, ↓ IFNα mRNA (cells)	(85, 86)
αIFNα mAb	↑ lethality	ND	(86)
αIFNβ mAb or *Ifnb^-/-^*	↑ lethality (100%)	V: ↑ viremia, ↑ tissue titers (some but not all tissues) I: no Δ Ab responses, no Δ brain infiltrate	(86, 87)
**Chikungunya virus, s.c. (footpad)**			
*Ifnar1^-/-^*	↑ lethality (100%)	V: ↑ viremia, ↑ tissue titers	(88, 89)
*Irf7^-/-^*	↑ foot swelling	V: ↑ viremia, ↑ tissue titers I: ↓ serum IFN, ↓ IFNα mRNA (tissue)	(90–91)
αIFNα mAb	↑ foot swelling	V: ↑ viremia, ↑ tissue titers	(90)
αIFNβ mAb or *Ifnb^-/-^*	↑ foot swelling	V: minimal Δ viremia and tissue titers I: ↑ neutrophil infiltrate (foot)	(90)
**Influenza A virus, PR/8/34 (H1N1), i.n.**			
B6.*Mx1.Ifnar1^-/-^* (functional Mx1 KI)	↑ lethality	ND	(92)
B6.*Mx1.Ifnb^-/-^* (functional Mx1 KI)	↑ lethality	V: ↑ lung titer	(92)
**Vaccinia virus, i.n.**			
*Ifnb^-/-^*	↑ weight loss, ↑ lethality	V: ↑ tissue titers	(93)
**Friend retrovirus, i.v.**			
*Ifnar1^-/-^*	ND	V: ↑ viremia, ↑ spleen titer I: ↓ CD4 T%, ↓ CD8 T% (spleen)	(94)
*Ifnb^-/-^*	ND	V: no Δ viremia, ↑ splenic titer I: ↓ CD4 T% (spleen)	(94)
rIFN α1, α4, α6, or α9 (B10.A×A.BY)F_1_	ND	V: ↓ viremia, ↓ spleen titer (α1, α4, α9); no Δ titers (α6) I: ↑ Ag^+^ CD8 T (α1 only), ↑ NK activation (α1, α4, α9)	(95)
rIFNα2, α5, or α11 (B6 or (B10.A×A.BY)F_1_)	ND	V: ↓ spleen titer (α11 only) I: ↑ NK activation	(96)
**Hepatitis B virus, hydrodynamic injection i.v.**			
rIFNα1, α2, α4, α5, α6, α9, or α11 (BALB/C)	ND	V: ↓ viremia (α4, α5); no Δ viremia (α1, α2, α6, α11) I: ↑ CTL and NK activity (α4, α5)	(97)
pIFNα, pIFNβ (hydrodynamic i.v.)	ND	V: ↓ viremia (pIFNα > pIFNβ) I: ↑ liver ISG induction (pIFNα > pIFNβ), no Δ T cell responses (pIFNα or pIFNβ)	(98)

The mouse genetic background is C57BL/6 unless otherwise specified.

↑, increased; ↓, decreased; Δ, change; αIFN, anti-IFN; Ag, antigen-specific; BALF, bronchoalveolar lavage fluid; CTL, cytotoxic lymphocyte (CD8 T cell); I, immune; i.n., intranasal; i.v., intravenous; ISG, interferon-regulated gene; KI, knock-in; mAb, monoclonal antibody; ND, no data; p, plasmid; r, recombinant; V, virological.

#### Lymphocytic Choriomeningitis Virus

Lymphocytic choriomeningitis virus (LCMV) is a nonlytic, negative-strand RNA virus and a prototypic member of the *Arenaviridae* family, which are causative agents of hemorrhagic fevers in humans (100). The host genetics, viral strain, dose, and inoculation route all have profound impacts on host responses and disease outcome, and this remains true for the role of type I IFN responses during LCMV pathogenesis (101). LCMV infection serves as an excellent example of the pathogenic potential of type I IFNs.

LCMV-Clone-13 (Cl-13), which differs from its parent strain LCMV-Armstrong (Arm) by just three amino acids, causes a persistent infection, whereas LCMV-Arm is acutely and effectively cleared by immunocompetent mice (102). A clear pathogenic role for type I IFNs during persistent LCMV-Cl-13 infection has been established (78, 79, 103–105). Loss of IFNAR1 caused increased viral loads early during infection but ultimately restored splenic organization, decreased expression of the negative immune regulators IL-10 and programmed death-ligand 1 (PD-L1), increased protective adaptive immune responses, and accelerated clearance of persistent virus (78, 79, 105). While both LCMV-Arm and LCMV-Cl-13 infection led to high IFNα levels in the serum, only LCMV-Cl-13 induced significant serum IFNβ (79). In a seminal study, Ng and colleagues showed that the pathogenic activity of type I IFNs in persistent LCMV infection could be ascribed to just one subtype—IFNβ. Using monoclonal antibody (mAb) blockade and genetic deletion, they showed that IFNβ was dispensable for controlling early LCMV-Cl-13 viral loads, suggesting that IFNα or other subtypes mediate these antiviral responses (80). Instead, blockade of IFNβ but not IFNα improved splenic architecture, decreased infection of CD8α^−^ DC, and enhanced antiviral T cell responses that led to clearance of persistent virus, mimicking many of the effects seen with IFNAR1 blockade. Altogether, persistent LCMV-Cl-13 infection serves as an important example that the type I IFN subtypes can have distinct properties *in vivo* that have profound impacts on viral pathogenesis.

As discussed above, LCMV-Cl-13 infection causes persistent infection in certain mouse strains (C57BL/6, BALB/C, C3H, or SWR/J); however, LCMV-Cl-13 infection of other strains (NZB, SJL/J, PL/J, NZO, or FVB/N mice) causes type I IFN- and CD8 T cell-dependent severe vascular leakage and death by about 6–8 days post infection (dpi) (81, 82, 106, 107). NZB.*Ifnar1* ^−/−^ but not NZB.*Ifnb* ^−/−^ mice were protected from LCMV-Cl-13 induced lethal vascular leakage, suggesting that IFNβ is dispensable for the detrimental effects of type I IFN in this model and that other subtypes like IFNα may drive this phenotype (81). However, this is challenged by the fact that blockade of IFNβ alone, pan-IFNα (α1, α4, α5, α11, and α13) alone, or combined pan-IFNα/β did not replicate the protection provided by anti-IFNAR1 treatment in FVB/N mice (82). The inability of IFNβ or IFNα blockade to phenocopy IFNAR1 blockade could be due to dosing issues, as the serum levels of IFNα were severely elevated (roughly 18-fold over IFNβ levels), involvement of IFNα subtypes not blocked by the mAb, or involvement other type I IFN subtypes altogether could be responsible for the lethal phenotype. Nevertheless, type I IFNs are clearly important host determinants of lethal LCMV infection, and the individual IFN subtype(s) responsible remains an open question.

#### Chikungunya and West Nile Viruses

Chikungunya virus (CHIKV) is a mosquito-transmitted, reemerging alphavirus that causes outbreaks of acute fever, rash, polyarthritis, arthralgia, and myositis (108). West Nile virus (WNV) is a mosquito-transmitted flavivirus that can cause encephalitis in severe cases (109). It is helpful to consider these models together because both models utilize a peripheral route of infection by inoculating the footpad subcutaneously (s.c.), and type I IFNs are essential for controlling both CHIKV and WNV, as *Ifnar1* ^−/−^ mice rapidly succumb to a severe, disseminated infection with either virus (83, 84, 88, 89). The collective evidence from these models suggest that IFNα and IFNβ play nonredundant protective roles.

Loss of IRF7, the master transcriptional regulator of IFNα subtypes, in acute WNV infection increased lethality and viral loads in both peripheral and central nervous system (CNS) tissues compared to WT animals (34, 85). Similarly, *Irf7* ^−/−^ mice infected with CHIKV developed worse clinical disease (foot swelling) and sustained high viral loads at the site of infection and sites of dissemination (90–92). The poor clinical outcome of *Irf7* ^−/−^ mice during WNV and CHIKV infection may be the result of decreased IFNα activity in the serum (85, 86, 91, 92). This postulation is supported by the observation that *Irf7* ^−/−^ mice produce little to no systemic IFNα activity when infected with a number of viruses, including Dengue virus (DENV), herpes simplex virus 1 (HSV-1), and encephalomyocarditis virus (EMCV), and this loss of systemic IFNα activity correlated with increased susceptibility to those infections (34, 110, 111). Pan-IFNα mAb blockade closely mimicked the clinical and virologic phenotype of *Irf7* ^−/−^ mice in CHIKV infection and phenocopied the lethality observed in WNV infection (86, 90). Altogether, these findings suggest that an important protective function of IRF7 is the production and amplification of IFNα responses and that IFNαs are important for controlling viral replication and dissemination.

In contrast with IFNα, the role of IFNβ *in vivo* is more varied and dependent on the biological context. *Ifnb* ^−/−^ mice are more susceptible than WT mice to WNV infection, and this increased lethality was accompanied with elevated viral burden in some but not all tissues (87). Specifically, WT and *Ifnb* ^−/−^ mice similarly controlled WNV replication in the spleen and serum, consistent with IFNα subtypes dominating serum IFN activity. WNV did replicate to a larger extent in the brain, spinal cord, and the draining lymph in *Ifnb* ^−/−^ mice compared to WT mice (87). An antiviral role for IFNβ has also been described for vaccinia virus and IAV infections (93, 94). In contrast to WNV infection, loss of IFNβ exacerbated CHIKV-induced disease but with minimal impact on viral burden at the inoculation site or distant tissues, suggesting that IFNβ may be important in restricting viral replication within certain but not all tissues (90). Rather, the increased disease severity of CHIKV-infected *Ifnb* ^−/−^ mice correlated with increased neutrophil accumulation at the site of infection, and depletion of neutrophils in *Ifnb* ^−/−^ mice reversed the disease exacerbation to WT levels. Altogether, these data from CHIKV and WNV infections point to the particular importance of IFNα subtypes in restricting viral replication and spread and highlight that the primary role of IFNβ varies depending on the specific context.

#### Human Immunodeficiency Virus 1 and Friend Retrovirus

Human immunodeficiency virus 1 (HIV-1) is a highly pathogenic retrovirus that leads to acquired immunodeficiency syndrome (AIDS). The relationship between type I IFNs and HIV-1 pathogenesis is complex, and it is outside the scope of the this review to cover all the protective and pathogenic functions, which have been extensively reviewed elsewhere (112–114). The purpose of reviewing HIV and Friend retrovirus (FV) infection is not to delve into whether type I IFNs have a net protective or pathogenic role, but rather, we seek to underscore that the IFNα subtypes are not equivalent in their antiviral or immunomodulatory properties *in vivo*.

Harper and colleagues evaluated the mRNA expression of specific IFNα subtypes in human pDCs following HIV-1 exposure (115). Intriguingly, they found an inverse relationship between the subtypes induced and their antiviral potency. HuIFNα1/13 and HuIFNα2 were highly expressed, but they demonstrated weaker antiviral activity *in vitro*, whereas HuIFNα6, α8, and α14 represented a smaller fraction of the IFNα subtypes induced but demonstrated the highest antiviral activity against HIV-1. Likewise, a study from Lavender and colleagues showed that therapeutic administration of HuIFNα14 was more beneficial than administration of HuIFNα2 in controlling HIV-1 replication in a humanized mouse model (116). The efficacy of IFNα14 was associated with increased ability to stimulate intrinsic immune responses including expression of tetherin and Mx2 as well as a greater frequency of TRAIL^+^ natural killer (NK) cells. Conversely, IFNα2 was superior in increasing the frequency of CD8^+^ T cells. An additional study used humanized mice that lack pDCs (Hu-PBL mice) and do not express much endogenous type I IFN during acute HIV-1 infection to study the impact of IFNα subtypes. They performed a single hydrodynamic injection of plasmid encoding different type I IFN subtypes (HuIFNα2, α6, α8, α14, or β) into Hu-PBL mice prior to HIV-1 infection (117). The authors found that all subtypes tested limited HIV-1 replication and prevented HIV-induced CD4^+^ T cell depletion by 10 dpi, but only HuIFNα14- and HuIFNβ-expressing mice demonstrated this protective effect out to 40 dpi. Altogether these findings demonstrate nonredundant functions of IFNα subtypes, with HuIFNα14 emerging as an intriguing subtype for further studies during HIV-1 infection.

Distinct properties of murine IFNα subtypes have also been observed in FV infection, a commonly used murine retrovirus model. A protective role for type I IFNs in controlling FV infection *in vivo* was demonstrated with *Ifnar1* ^−/−^ and *Ifnb* ^−/−^ mice both having increased viral loads in the spleen. However, only *Ifnar1* ^−/−^ mice showed a significant increase in viremia (95). These findings suggest that both IFNα and IFNβ protect against FV infection, but IFNα may be more important for controlling systemic infection and dissemination. Different potencies among IFNα subtypes have also been revealed. *Ex vivo* stimulation of FV-specific CD8^+^ T cells demonstrated differential activities among the IFNα subtypes. IFNα4, α6, and α9 had the strongest effects on CD8^+^ T cells, including inhibiting proliferation, stimulating cytokine production, and enhancing cytotoxicity (118). Treatment of FV-infected mice with MuIFNα1, α4, or α9, but not α6, significantly decreased viral loads, and subtype effectiveness was associated with different mechanisms (96). Only IFNα1 treatment correlated with activated FV-specific CD8^+^ T cells in the spleen, whereas NK cell activation was observed after treatment with all examined IFNα subtypes. Another study demonstrated that prophylactic administration of MuIFNα11, but not α2 or α5, significantly reduced viral loads by activating NK cells and ultimately provided long-term protection (6 weeks) (97). Together with the HIV-1 studies, retroviruses have proven to be effective tools for probing the diverse functions IFNα subtypes.

#### Hepatitis B and Hepatitis C Viruses

Hepatitis B (HBV) and hepatitis C viruses (HCV) are drastically distinct pathogens from a virological perspective—HBV is a double-stranded DNA virus belonging to the *Hepadnaviridae* family, whereas HCV is a positive-strand RNA virus and a member of *Flaviviridae*. However, both viruses display tropism for hepatocytes, and chronic infection with either virus can lead to liver failure, cirrhosis, and hepatocellular carcinoma (119). Beginning in the 1980s, derivatives of recombinant HuIFNα2 were used to treat chronic HBV and HCV, but treatment was successful in a limited subset of patients and severe side effects were common [reviewed in reference (120)]. These issues have led to the phasing out of type I IFN-based therapeutics in favor of direct-acting antiviral drugs (120). Though HuIFNα2-based therapeutics are the only approved type I IFN therapies for HCV or HBV treatment, pilot studies of IFNβ therapy in IFNα-nonresponding HBV or HCV patients suggest some beneficial effects of IFNβ as well (121–123). These findings suggest that other IFN subtypes in addition to IFNα2 may offer protective effects against hepatitis viruses.

Indeed, one study with the HBV hydrodynamic injection model demonstrated that prophylactic treatment with MuIFNα4 or α5 was more effective than other IFNα subtypes in decreasing HBV replication *in vivo*, and both α4 or α5 also increased effector NK and CD8^+^ T cell frequencies in the liver and spleen (98). Hydrodynamic injection of plasmids expressing MuIFNα4, α5, or combined α4 and α5 was more effective than treatment with the respective recombinant proteins, highlighting the importance of long-lasting endogenous IFNα expression in the liver during HBV infection. Another study directly showed differential effects of IFNα4 and IFNβ in the hydrodynamic injection HBV model (99). Co-injection of a plasmid encoding MuIFNα4 with HBV DNA decreased HBV serum markers, elevated liver ISG expression, and reduced HBV^+^ cells in the liver, whereas co-injection of an IFNβ-expressing plasmid demonstrated weaker inhibition of HBV and surprisingly led to a transient increase in HBV^+^ hepatocytes. This increase in HBV^+^ hepatocytes was not observed if the IFNβ plasmid was injected 14 dpi instead of co-injected with HBV (99). Even as the currently approved type I IFN therapies are being phased out of clinical use, these findings add to the accumulating evidence of distinct potencies and functions of IFNα and IFNβ subtypes in mouse models of relevant human pathogens.

#### IFNω Subtype Differences

IFNω is understudied compared to IFNα/β subtypes likely because mice lack a functional IFNω, but there is much *in vitro* evidence that it signals and functions similarly to IFNα/β (61, 124). Humans have only one IFNω subtype, but several species possess an expansion of IFNω genes (15–17, 125, 126). A number of these IFNω subtypes have been cloned from several species and have been demonstrated to be functional type I IFNs (127–129). Just as there is growing appreciation that expanded IFNα subtypes provide an evolutionary advantage beyond redundancy, it stands to reason that the expansion of IFNω genes likewise imparts a fitness advantage for those species. Indeed, a recent study compared two different IFNω subtypes from *Rousettus aegyptiacus* bats and found that IFNω9 displayed more effective antiviral activity against several RNA viruses *in vitro* compared to IFNω4 (130). Additionally, differences in expression and activity of porcine IFNω subtypes have also been demonstrated, with IFNω7 demonstrating the best antiviral activity *in vitro (* 131). Several of these animals with expanded IFNω subtypes represent important reservoirs and transmitters of relevant human pathogens, so IFNω functional studies may provide valuable information on understanding the interactions between pathogens and their natural hosts.

#### Remarks on Viral Infections

When type I IFNs act on the proper cell type at the opportune time, they can induce an antiviral state, promote apoptosis of virally infected cells, coordinate recruitment of immune cells, enhance activation of antigen-presenting cells, and augment protective B and T cell responses. Not all IFNs are equal in their ability to induce these protective effects, and exploring this idea *in vivo* is an active area of research. Studies from infection with LCMV, WNV, and CHIKV have made it evident that endogenous IFNα subtypes are particularly important for limiting viremia and viral spread, likely due to their abundant activity in the serum in a number of viral infections. In peripheral tissues, IFNαs and IFNβ can exert important antiviral or immunomodulatory activity. Whether a particular subtype emerges as more important than others is likely going to depend on its biochemical properties, the cellular tropism of the virus, the source and magnitude of its induction, how long its expression is sustained, and the specific cell types responding to IFN.

If type I IFN signaling is sustained too long, immunosuppression and viral persistence can occur through the upregulation of negative immune regulators, like IL-10 and PD-L1. LCMV infection is a good example of this scenario, and strikingly, IFNβ was critical in promoting many detrimental features of type I IFN signaling in this model. We did not have space to discuss the growing evidence that type I IFNs can promote tissue damage during acute viral infections by promoting excessive inflammation and cell death [discussed in references (132, 133)]. This has been observed for mouse strains highly susceptible to influenza or coronavirus infection (134–136). The mechanisms responsible for these detrimental effects of type I IFN are an active area of research, but initial observations suggest that excessive or delayed IFN induction may play a role. It is also unknown whether specific IFN subtypes are responsible for these effects. Future studies exploring this possibility could have an important impact on human disease.

### Bacterial Infections

Type I IFNs can play a pathogenic or protective role during bacterial infection depending on the pathogen. The mechanisms underlying the beneficial or detrimental roles during bacterial infection remain poorly understood and warrant further study. Below we explore some of the properties of type I IFNs during models of bacterial infections (**Table 2**). However, compared to the examples from viral infections, few of these studies directly compare the functions of IFNα and IFNβ. We draw attention to a few instances in which specific subtypes have been examined and highlight areas where this may be an interesting avenue to explore.

**Table 2:** Summary of IFNα and IFNβ functions in mouse models of bacterial infections.

Intervention	Clinical Outcome	Bacterial Load and Immune Characterization	Refs.
***Mycobacterium tuberculosis (Mtb)***			
129.*Ifnar1^-/-^ Mtb* (H37Rv)	↓ lethality	B: ↓ lung titer I: ↓ iNOS expression (lung), ↓ IL-1β, IL-1α, IL-6 (lung)	(137)
129.*Ifnar1^-/-^ Mtb* (HN878)	↓ lethality	B: ↓ lung titer	(138)
*Ifnar1^-/-^* or B6.*Sst1^S^.Ifnar1^-/-^ Mtb* (Erdman)	↓ lethality	B: ↓ lung titer I: ↓ IL-1Ra (lung), ↑ functional IL-1β activity (lung)	(139, 140)
*Ifnar1^-/-^ Mtb* (H37Rv)	ND	B: ↓ lung titer I: ↑ IL-1α, IL-1β expression (lung myeloid cells *in vivo*), ↑ PGE2 in BALF	(141, 142)
Poly-ICLC (i.n.), *Mtb* (H37Rv)	↑ lethality, ↑ lung necrosis IFNAR1	B: ↑ lung titer (acute, chronic) I: ↑ CD11b^+^F4/80^+^GR1^int^ infiltrate (lung)	(142, 143)
***Salmonella enterica* serovar Typhimurium**			
*Ifnar1^-/-^* adult (i.v.)	↓ lethality	B: ↓ spleen CFU I: ↑ Mφ freq. (spleen), ↓ Mφ cell death (spleen)	(144)
*Ifnb^-/-^* adult (oral)	↓ lethality	B: ↓ liver CFU I: ↓ IL-10 mRNA, ↑ CXCL2 mRNA, ↑ MPO activity (small bowel)	(145)
***Streptococci* s*pp.***			
*Ifnar1^-/-^ S. pyogenes*, s.c.	↑ lethality	B: ND I: ↑ neutrophil infiltrate (lung)	(146)
129.*Ifnar1^-/-^* (adult) Group B, type V, i.p.	↑ lethality	B: ↑ blood and kidney CFU	(147)
*Ifnb^-/-^* (adult) Group B, type V, i.p.	↑ lethality	B: ND I: ↓ TNFα and IFNγ induction by peritoneal Mφ (*ex vivo*)	(147)
129.*Ifnar1^-/-^ S. pneumoniae*, i.v. or i.c.	↑ lethality	B: ↑ blood CFU (i.v. and i.c. routes)	(147)
*Ifnar1^-/-^ S. pneumoniae*, i.n. or i.p.	ND	B: ↑ blood CFU (i.n. route), no Δ viremia (i.p. route) I: ↑ lung permeability, ↓ tight junction mRNA (lung)	(148)
rIFNβ (i.n.), *S. pneumoniae*, i.n.	↓ lethality	B: ↓ blood CFU	(148)
AdIFNα (i.n.), *S. pneumoniae*, i.n.	↓ lethality	B: ↓ lung, ↓ spleen CFU I: ↓ neutrophil and Mφ infiltrate (lung), ↓ BALF TNFα, IL-1β, and CXCL10	(149)
***Listeria monocytogenes***			
*Ifnar1^-/-^* (various routes)	↓ lethality	B: ↓ liver, ↓ spleen CFU I: ↓ TRAIL expression (spleen), ↓ apoptosis (spleen), ↑ serum IL-12p70, ↓ serum TNFα and IL-6	(150–151)
*Irf3^-/-^* (i.v.)	↓ lethality	B: ↓ liver, ↓ spleen CFU I: ↓ IFNβ induction in Mφ (*ex vivo*), ↓ apoptosis (spleen)	(152)

The mouse genetic background is C57BL/6 unless otherwise specified.

↑, increased; ↓, decreased; Δ, change; Ad, adenoviral vector expression; B, bacterial load; BALF, bronchoalveolar lavage fluid; CFU, colony forming unit; dep., dependent; freq., frequency I, immune; i.c., intracranial; i.v., intravenous; Mφ, macrophage; MPO, myeloperoxidase; ND, no data; Poly-ICLC, polyinosinic-polycytidylic acid stabilized with poly-_L_-lysine; s.c., subcutaneous; spp., species.

#### Mycobacterium Tuberculosis

*Mycobacterium tuberculosis* (*Mtb*) causes the disease tuberculosis and represents a global health burden. This intracellular pathogen primarily infects the lungs, and it can enter latency if it is not eliminated, persisting in granulomas (154). The actions of type I IFNs during *Mtb* infections are complex, and there are numerous examples of contradictory findings. Overall, there is strong evidence that type I IFNs are detrimental to the host, but depending on the timing of IFN induction, the bacterial strain, and host genetics, IFNs may occasionally benefit the host during infection [reviewed in reference (155)].

Numerous studies have shown a type I IFN-inducible transcriptional profile in blood isolated from patients with active tuberculosis, but this signature is typically absent in patients with latent infection or patients who have undergone successful treatment (156–158). Concordantly, infection with hypervirulent *Mtb* laboratory strains showed increased recruitment of type I IFN-producing pDCs and classical DCs and elevated expression of IFNα or IFNβ in the lung, depending on the study (138, 139, 159–162). Multiple studies with human and mouse models have shown that type I IFNs are associated with impaired IFNγ-mediated antibacterial effects, decreased expression of IL-1α and IL-1β, decreased production of prostaglandin E2 (PGE2), and upregulation of IL-10 (138–142, 159, 162–165). Type I IFNs are also associated with increased cell death of macrophages and increased recruitment of myeloid cells permissive to *Mtb* infection (137, 143). Limited work has addressed the pathogenic potential of individual type I IFNs, but one recent study found that *in vitro* blockade of IFNα (subtypes unspecified), but not IFNβ blockade, significantly decreased intracellular *Mtb* bacterial load in a macrophage cell line (166). It remains to be determined if a similar effect could be observed *in vivo*.

Despite all of the evidence pointing to detrimental effects of type I IFNs in *Mtb* infection, type I IFNs may play a beneficial role in particular circumstances. First, several case reports have suggested that coadministration of IFNα with antimycobacterial therapy decreased bacterial burden in individuals who failed to respond to antimycobacterial therapy alone (167–170). However, these studies were employed before the pathogenic effects of type I IFNs were appreciated, and the mechanisms driving the apparent protection remain elusive. Second, in agreement with the findings that the detrimental effects of type I IFNs are largely due to inhibition of IFNγ, type I IFNs appear to be protective in contexts of IFNγ deficiency. Mice lacking both type I and type II IFN receptors displayed increased mortality and pathology compared to mice lacking only the type II IFN receptor in *Mtb* infection (171, 172). Mechanistically, type I IFNs may dampen recruitment of *Mtb-*permissible macrophages and suppress macrophages from entering an alternative activation state. In accord with these mice studies, administration of IFNα2b combined with antimycobacterial chemotherapy had beneficial effects in *Mtb*-infected children with underlying IFNγ signaling deficiencies (173, 174). It is unclear whether IFNβ can induce these effects as well. Further head-to-head comparison studies of IFNα and IFNβ are needed to determine if this protective effect of type I IFNs is unique to IFNα.

Type I IFNs may also benefit the host in infection with less virulent *Mycobacterium* strains, such as the bacille Calmette-Guérin (BCG) vaccine derived from *M. bovis (* 175, 176). Administration of IFNα at the time of BCG vaccination (s.c.) in mice followed by intramuscular IFNα boosts (subtype not disclosed) promoted production of IFNγ, tumor necrosis factor (TNF), and IL-12, thus slightly increasing the protection seen upon re-challenge with *Mtb* intranasal (i.n.) compared to immunization with BCG alone (175). Moreover, the bacterial ESX-1 secretion system promotes type I IFN induction, and its recombinant expression in the BCG vaccine better protected against *Mtb* infection than other versions of the vaccine (176–179). *In vitro* data also highlight the complexity of type I IFN functions, as pretreatment of permissible cells with IFN before *Mycobacterium* infection can promote bacterial growth or increase immune activation, depending on the cell type and bacterial strain (180, 181). Thus, type I IFNs may play a protective role in vaccination with weaker *Mycobacterium* strains.

#### Salmonella enterica Serovar Typhimurium

*Salmonella* is a common, pathogenic genus of bacteria that causes acute gastroenteritis. Type I IFNs largely play a pathogenic role in *Salmonella* infection by promoting necroptosis and suppressing protective innate cell recruitment and proinflammatory responses. Deletion of IFNAR1 increased survival of adult mice infected (i.v.) with *S. enterica* serovar Typhimurium (*S.* Typhimurium) and decreased splenic bacterial loads (144). Additionally, splenic macrophages in *Ifnar1* ^−/−^ mice were resistant to *S.* Typhimurium-induced necroptosis *ex vivo*, and a follow-up mechanistic study further determined that type I IFN signaling impaired antioxidative stress responses to *S.* Typhimurium infection of bone marrow-derived macrophages (144, 182). IFNβ may be the dominant type I IFN subtype driving this necroptosis phenotype, as blockade of IFNβ, but not IFNα, prevented necroptosis and enhanced survival of bone marrow-derived macrophages during *S.* Typhimurium infection *in vitro (* 144). It is unclear how many IFNα subtypes the antibody used blocks (clone: RMMA-1), so it is premature to rule out a contribution of IFNα. A role for IFNβ was further demonstrated in a separate study which showed that *Ifnb* ^−/−^ mice were more resistant to oral infection of *S.* Typhimurium, which was characterized by decreased bacterial burden, dampened expression of IL-10, and increased levels of CXCL2 and myeloperoxidase activity in the liver (145). Altogether, these findings suggest that IFNβ may play a detrimental role in *S.* Typhimurium infection by negatively regulating protective immune responses, but further studies are needed to rule out the involvement of other type I IFN subtypes.

#### Listeria monocytogenes

*Listeria monocytogenes* is an intracellular, pathogenic bacteria that causes sepsis and meningitis in immunocompromised and pregnant individuals (183). Many groups have shown that type I IFN signaling is detrimental to the host in systemic *L. monocytogenes* infection, but not in all routes of infection (150–153, 184, 185). Despite the important role that type I IFNs play in *L. monocytogenes* pathogenesis, the contribution of individual subtypes remains unknown. *Irf3* ^−/−^ mice displayed increased resistance to *L. monocytogenes* infection (60% survival), which almost phenocopied the resistance seen in *Ifnar1* ^−/−^ mice (80% survival) (152). Additionally, C57BL/6ByJ mice, which have a polymorphism in *Irf3* causing inefficient splicing of its mRNA, demonstrated reduced IFNβ induction and increased resistance to *Mtb* infection (186). These observations may suggest an important role for IFNβ in susceptibility to *L. monocytogenes* infection. However, these studies did not assess IFNα induction, and characterization of *Ifnb* ^−/−^ mice is needed to confirm this hypothesis. Mechanistically, loss of type I IFN attenuated *Listeria*-induced cell death in myeloid cells and lymphocytes *in vivo* and *ex vivo (* 150, 152, 187, 188). Antigen-stimulated T cells were more sensitive to lysteriolysin O (LLO)-induced apoptosis after exposure to IFNα compared to cells only treated with LLO (150). Thus, a role for IFNα subtypes should not be discounted. Altogether, it is impossible to draw firm conclusions about the roles of individual type I IFNs in *L. monocytogenes* infection with the currently available information. Studies that specifically block IFNα or IFNβ in *Listeria* infection might yield important insight into the functions of type I IFN subtypes.

#### Streptococci Species

*Streptococci* species often colonize mucosal surfaces and skin of healthy individuals without causing disease, but they can cause a variety of serious diseases in immunocompromised individuals or newborns (189). Type I IFNs appear to play a protective role during infection with a variety of *Streptococci* species (146–149).

*S. pneumoniae*, an alpha-hemolytic species commonly known as pneumococcus, is an opportunistic pathogen that colonizes the mucosal surfaces of the upper respiratory tract and is a leading bacterial cause of otitis media, pneumonia, sepsis, and meningitis (190). Type I IFNs play a beneficial role during pneumococcal infection, though the route of infection matters (147, 148). Loss of IFNAR1 increased lung permeability by decreasing tight junction protein expression, which is consistent with increased bacterial titer in the blood if *S. pneumoniae* was inoculated *via* an i.n. route but not *via* an intraperitoneal (i.p.) route (148). IFNβ played a role in mediating these protective effects because pre-treatment of mice with recombinant IFNβ i.n. significantly increased survival following *S. pneumoniae* challenge and decreased blood bacterial titer. However, IFNα subtypes likely provide beneficial effects as well since a separate study showed that prophylactic or therapeutic administration (i.n.) of an adenoviral vector expressing IFNα enhanced survival after pneumococcal infection and decreased lung and spleen bacterial burden (149). It is unclear which IFNα subtype was used in this study, so more work is needed to determine if some IFNα subtypes are more potent than others.

A protective role of type I IFNs was also demonstrated in infection with the beta-hemolytic species *S. pyogenes* (group A streptococcus, GAS) and *S. agalactiae* (group B streptococcus, GBS) (146, 147). In GBS i.v. challenge, IFNβ transcript was more robustly induced in the spleen compared to IFNα4, and *Ifnb* ^−/−^ mice demonstrated increased lethality compared to WT mice (147). Additionally, *in vitro* GBS infection poorly activated peritoneal macrophages from *Ifnar1* ^−/−^ or *Ifnb* ^−/−^ mice compared to WT controls, suggesting that IFNβ may function to augment macrophage antibacterial properties. However, carefully controlled experiments need to be performed in order to determine if IFNβ is directly modulating macrophage activation or if IFNβ acts indirectly by influencing bacterial loads. The role of specific subtypes was not evaluated in GAS infection; however, macrophages and DCs were found to induce IFNβ downstream of unique pathways. Macrophages required IRF3, STING, TBK1, MyD88, and stimulation with streptococcal DNA, whereas DCs depended on MyD88, IRF5, and streptococcal RNA (146). It might be interesting to evaluate *Irf3* ^−/−^ *, Irf5* ^−/−^, and *Ifnb* ^−/−^ mice in *S. pyogenes* infection to determine if the cellular source of IFN affects pathogenesis. Additionally, better characterization of the IFNα subtypes induced and their role in GAS and GBS is needed.

#### Remarks on Bacterial Infections

Similar to viral infections, type I IFNs can be either detrimental or beneficial to the host during bacterial infections, depending on the specific pathogen. The mechanisms underlying these divergent outcomes share many features with viral infections. The ability of type I IFNs to regulate cell death, suppress protective IFNγ responses, and/or upregulate IL-10 can account for the detrimental functions of type I IFNs during *Mtb*, *Salmonella*, and *L. monocytogenes* infection. These activities are reminiscent of the type I IFN-driven increases in IL-10 and PD-L1 observed in LCMV, as well as the increased cell death observed in acute influenza infection (132, 135). Even though a detrimental role for type I IFNs is well documented in *Mtb* infection, in special contexts type I IFNs may be able to serve a protective function. Of particular interest is the possibility of type I IFN serving as an adjuvant with certain, less virulent *Mycobacterium* vaccination strains. As is the case with some viral infections, the timing, magnitude, and cellular source of type I IFNs underlie these distinct outcomes. In the future it will be interesting to explore if these divergent phenomena are also due to differential induction or functions of type I IFN subtypes.

There are also examples of type I IFNs having a protective role in bacterial infections, such as with several *Streptococcus* species. This net beneficial effect may reflect many of the functions commonly observed in viral infections, such as coordinating protective immune cell recruitment and activation and promoting the right level of inflammation needed to clear the bacterial infection. The exact mechanisms underlying these protective effects are understood at a very general level and questions remain. Which cells do IFNs signal on to mediate these protective effects? What ISGs are responsible for mediating protection, and are they different from those acting in viral infections? Importantly, do specific type I IFN subtypes drive particular protective functions? We are only beginning to grasp how type I IFNs contribute to protective antibacterial immune responses, and there are many interesting avenues to explore relevant to human health.

### Parasitic Infections

Parasites include single-cellular protozoa (e.g. *Plasmodium* and *Leishmania* species) and multicellular helminths, which include flatworms (e.g. *Schistosoma* species) and roundworms (e.g. *Ascaris* species) (191–194). Previously, parasite-host interaction studies have not investigated the functions of type I IFNs, but recent studies in malaria have identified both protective and pathogenic properties of IFNα/β [reviewed in references (195, 196)]. Below we explore the roles of IFNα and IFNβ during *Plasmodium* infection, the causative agent of malaria (**Table 3**).

**Table 3:** Summary of IFNα and IFNβ functions in mouse models of malaria infection.

Intervention	Clinical Outcome	Bacterial Load and Immune Characterization	Refs.
**Liver-stage**			
*Ifnar1^-/-^ P. berghei* (ANKA) (early time points)	ND	P: ↑ parasitemia, ↑ liver titer I: ↓ ISG induction (liver)	(197)
*Irf3^-/-^ P. berghei* (ANKA) (early time points)	ND	P: ↑ liver titer I: ↓ ISG induction (liver)	(197)
*Irf7^-/-^ P. berghei* (ANKA) (early time points)	ND	P: ND I: ↓ ISG induction (liver)	(197)
*Ifnar1^-/-^ P. yoelii* (Py17XNL)	ND	P: ↑ liver titer (bioluminescence) I: ↓ NKT cells (liver); no Δ NK, CD4, and CD8 T cells (liver)	(198)
*Irf3^-/-^ P. yoelii* (Py17XNL)	ND	P: ↑ liver titer (bioluminescence)	(198)
*Irf7^-/-^ P. yoelii* (Py17XNL)	ND	P: no Δ liver titer (bioluminescence)	(198)
**Blood-stage**			
*Ifnar1^-/-^ P. chabaudi*	ND	P: ↓ parasitemia I: ↑ serum IFNγ	(199)
*Irf7^-/-^ P. chabaudi*	ND	P: ↓ parasitemia I: ↑ serum IFNγ, ↑ IFNγ^+^ CD4 T freq. (spleen)	(199)
*Ifnar1^-/-^ P. yoelii* (Py17XNL)	ND	P: ↓ parasitemia (late) I: ↑ serum Ab titer, ↑ Tfh cells and GC B cells (spleen)	(200)
rIFNα (18 hpi, i.v.), lethal *P. yoelii* (YM)	↓ lethality (0%)	P: ↓ parasitemia	(201)
rIFNα1/α8 (i.p.), *P. yoelii* (265 BY)	ND	P: ↓ parasitemia I: no Δ RBC count, ↓ reticulocytosis	(202)
rIFNα1/α8 (i.p.), *P. yoelii* (Py17XNL)	ND	P: ↓ parasitemia (early); trend ↑ parasitemia (late)	(202)
**Cerebral-stage**			
*Ifnar1^-/-^ P. berghei* (ANKA)	↓ lethality (0%); ↓ cerebral hemorrhage	P: ↓ parasitemia (variable); ↓ brain titer I: ↑ serum IFNγ, ↑ IFNγ^+^ CD4 T (brain, liver, spleen); ↓ CD8 T infiltrate (brain), ↓ BBB leakage	(199, 203–207)
*Irf3^-/-^ Irf7^-/-^ P. berghei* (ANKA)	↓ lethality (0%)	P: ND	(203)
*Irf7^-/-^ P. berghei* (ANKA)	↓ lethality	P: ↓ parasitemia, no Δ brain titer I: ↓ CD8 T infiltrate (brain)	(199)
rIFNβ (i.p.), *P. berghei* (ANKA)	↓ lethality	P: ND I: ↓ BBB leakage, ↓ CXCL9 (brain), ↑ CXCL10 (brain), ↓ T cell infiltrate (brain)	(208)
rIFNα1/α8 (i.p.), *P. berghei* (ANKA)	↓ lethality	P: ↓ parasitemia, ↓ brain titer I: ↓ Mφ, neutrophil, CD4 T, and CD8 T infiltrate (brain)	(209)

The mouse genetic background is C57BL/6 unless otherwise specified.

↑, increased; ↓, decreased; Δ, change; Ab, antibody; BBB, blood brain barrier; freq., frequency; GC, germinal center; hpi, hours post infection; I, immune; i.p., intraperitoneal; i.v., intravenous; LN, lymph node; Mφ, macrophage; ND, no data; P, parasite; RBC, red blood cell; Tfh, T follicular helper.

#### Plasmodium Overview

Malaria initially presents as a wide variety of symptoms, including periodic fever, chills, headache, malaise, and muscle and joint aches, but as disease progresses severe anemia, blood acidosis, splenomegaly, acute respiratory distress syndrome, and spread to the brain are possible, which can be fatal (210). Infected mosquitoes transmit *Plasmodium* sporozoites to humans during a blood meal. The sporozoites initially infect hepatocytes, where they replicate as merozoites (liver stage), and eventually, merozoites enter the blood stream to infect red blood cells, where they begin asexual reproduction (blood stage) (191). Symptoms in humans usually begin developing several days after release of parasites into the blood. *P. falciparum* and *P. vivax* are the most common species responsible for malaria disease in humans, and several *Plasmodium* species (*P. berghei, P. yoelii, P. chabaudi*, and *P. vinckei*) infect rodents and recapitulate various stages of human disease (210).

#### Liver-Stage Malaria

Two important studies recently revealed a protective role for type I IFNs in controlling liver-stage *Plasmodium* infection. First, Liehl and colleagues showed that all of the early upregulated genes in the liver from mice infected with *P. berghei* (ANKA) were classified as IFN-stimulated genes or linked to the type I IFN signaling pathway (197). Similarly, Miller *et al.* also uncovered an early type I IFN signature in the liver of mice infected with *P. yoelii* (Py17XNL) (198). Upon global IFNAR1 deficiency or conditional deletion of IFNAR1 on hepatocytes (Albumin-Cre), mice failed to control parasite replication in the liver (197, 198). These studies suggest that type I IFN signaling protects against malaria infection by controlling early parasite replication in the liver. Further characterization revealed that *Irf3* ^−/−^ mice, but not *Irf7* ^−/−^ mice, showed a similar early increase in liver parasite burden as *Ifnar1* ^−/−^ mice following *P. yoelii* (Py17XNL) infection (198). This is consistent with the observation that *Irf3* ^−/−^ mice demonstrated a more severe decrease in early liver ISG induction compared to *Irf7* ^−/−^ mice following *P. berghei* (ANKA) infection (197). Given that IRF3 is a key regulator of IFNβ induction, these findings could suggest that endogenous IFNβ is more important than IFNα subtypes for controlling parasite burden in liver stage malaria. Additional studies are needed to confirm this hypothesis.

#### Blood-Stage Malaria

There is conflicting evidence for whether type I IFNs have a net beneficial or detrimental effect during the blood stage of malaria. Evidence for a protective role is as follows. First, treatment of mice with recombinant hybrid HuIFNα1/α8, which has activity on murine cells, concurrent with *P. yoelii* (265 BY) infection decreased early parasitemia, and the authors proposed that this was due to IFNα-dependent inhibition of reticulocyte (immature red blood cell) development, as opposed to direct anti-plasmodium effects (202, 211). Moreover, deletion of inflammasome components or some intracellular PRR sensing components decreased parasitemia and increased resistance to lethal *P. yoelii* infection through alleviation of SOCS1-mediated suppression of type I IFN responses (201, 212).

Other studies have demonstrated that type I IFNs might play a detrimental role during blood-stage malaria. First, a group showed that *Ifnar1* ^−/−^ and *Irf7* ^−/−^ mice better controlled parasitemia in non-lethal *P. chabaudi* infection compared to WT controls (199). Additionally, Sebina and colleagues showed that IFNAR1 deletion in *P. yoelii* (Py17XNL) infection increased pathogen-specific antibody titers and decreased parasitemia late in infection (17–21 dpi) (200). Mechanistically, type I IFN signaled on DCs to limit their activation of T follicular helper cells in an inducible T cell co-stimulator (ICOS) signaling-dependent manner, and this interaction ultimately influenced downstream germinal center B cell responses (200). However, it should be noted that IFNAR1 deletion in the Sebina *et al.* study also trended toward increased parasitemia early in infection (6–11 dpi), suggesting that these findings are not completely incongruous with the studies that found a protective role for type I IFNs. Altogether, type I IFNs might be detrimental in the blood stage malaria by impeding humoral immunity later in infection, but the *Plasmodium* strain and timing of IFN action may influence the overall effect of type I IFNs on disease outcome. It would be interesting to determine if this effect is dependent on certain type I IFN subtypes.

#### Cerebral Malaria

Similar to the blood stage, the role of type I IFNs during cerebral malaria remains controversial. Several independent groups have demonstrated that *Ifnar1* ^−/−^ mice are either completely or partially protected from lethal experimental cerebral malaria (*P. berghei* ANKA sporozoite infection), demonstrating a net pathogenic effect for type I IFNs in this context (199, 203–207). Loss of type I IFN signaling may increase IFNγ-producing CD4^+^ T cells, reduce pathogenic CD8^+^ T cell recruitment and/or activation in the brain, improve DC priming of CD4^+^ T cell responses, or some combination thereof (199, 204–207). *Irf7* ^−/−^ mice only partially recapitulated the decreased brain pathology and protection from *P. berghei* (ANKA) lethality observed in *Ifnar1* ^−/−^ mice, but loss of IRF7 perfectly phenocopied the decreased parasitemia observed in *Ifnar1* ^−/−^ mice (199). These findings may suggest IFNαs are more important in promoting parasitemia, whereas IFNβ and IFNα might both contribute to brain pathology, but specific antibody blockade of type I IFN subtypes would confirm this hypothesis.

Paradoxically, a few groups have shown that systemically administering either recombinant IFNβ or hybrid IFNα1/α8 concurrently with infection alleviated cerebral malaria (*P. berghei* ANKA) (208, 209). Both IFN treatments reduced parasite burden in the brain and decreased infiltrating CD8^+^ T cells in the brain compared to control mice, but only IFNα1/α8 treatment decreased blood parasitemia (208, 209). A more recent study identified receptor transporter protein 4 (RTP4) as a positive regulator of type I IFN responses, and *Rtp4* ^−/−^ mice were completely protected from *P. berghei* (ANKA) lethality and brain pathology (213). This protection in *Rtp4* ^−/−^ mice correlated with increased type I IFN responses in microglia isolated from the brain, suggesting a protective role for IFNs, but blockade of type I IFN signaling in *Rtp4* ^−/−^ mice is needed to confirm a causal link (213). Overall, an issue of magnitude and timing of IFN response might underlie these apparent discrepancies with the protective phenotypes of *Ifnar1* ^−/−^ mice (discussed below). Indeed, antibody blockade of IFNAR1 as late as 5 dpi was almost as protective as *Ifnar1* ^−/−^ mice, suggesting that the detrimental effects of type I IFNs occurred during priming of adaptive immune responses (199).

#### Remarks on Parasitic Infections

It is clear that the role of type I IFNs in malaria is complex and depends on the stage of *Plasmodium* life cycle. Type I IFNs seem to play a protective role during the liver stage, but there are contradictory findings from various models of blood-stage and cerebral malaria. Perhaps infection with some strains of *Plasmodium* yields suboptimal type I IFN production very early in infection, ultimately leading to delayed and higher levels later in infection when parasite burden is not effectively controlled. Proper intervention at either step would benefit the host, and this could explain why loss of IFN signaling or exogenous IFN treatment can both be protective. The contribution of individual IFN subtypes remains unclear, though divergent phenotypes in *Irf3* ^−/−^ and *Irf7* ^−/−^ mice suggest this could be an interesting question to explore. Importantly, genetic variants in *IFNAR1* have been associated with either greater or lower risk of severe malaria disease (205, 214–217). The impact of each genetic variant on IFNAR1 expression and function still need to be determined, but these findings suggest that type I IFNs are important regulators of malaria disease in humans.

Overall, parasitic pathogens are biologically very diverse, so data from other parasitic infection models are needed to begin drawing broad conclusions. A recent study demonstrated that the TLR4-IRF1-IFNβ axis played a protective role in mice infected with *Leishmania infantum* by dampening proinflammatory pathways and IFNγ production by CD4^+^ T cells (218). RNA sequencing analysis of human samples revealed that upregulation of TLR4 and type I IFN pathways was associated with asymptomatic individuals compared to patients with visceral leishmaniasis (218). Another group found that *Ifnar1* ^−/−^ mice were more susceptible to *Toxoplasma gondii* infection (219). It would be interesting to know if IFNs are generally more important in single-cellular parasitic infections. That said, the multicellular helminth *Schistosoma mansoni* can induce a systemic type I IFN signature in mice and activate TLR3 in DCs *in vitro*, suggesting that a role for type I IFNs in parasitic worm infections is certainly possible (220, 221). Continued work to delineate the cellular sources and functions of type I IFNs in malaria and other parasitic diseases may reveal novel opportunities for therapeutic intervention and help uncover novel functions of type I IFNs.

## Cancer

**Figure 2:**
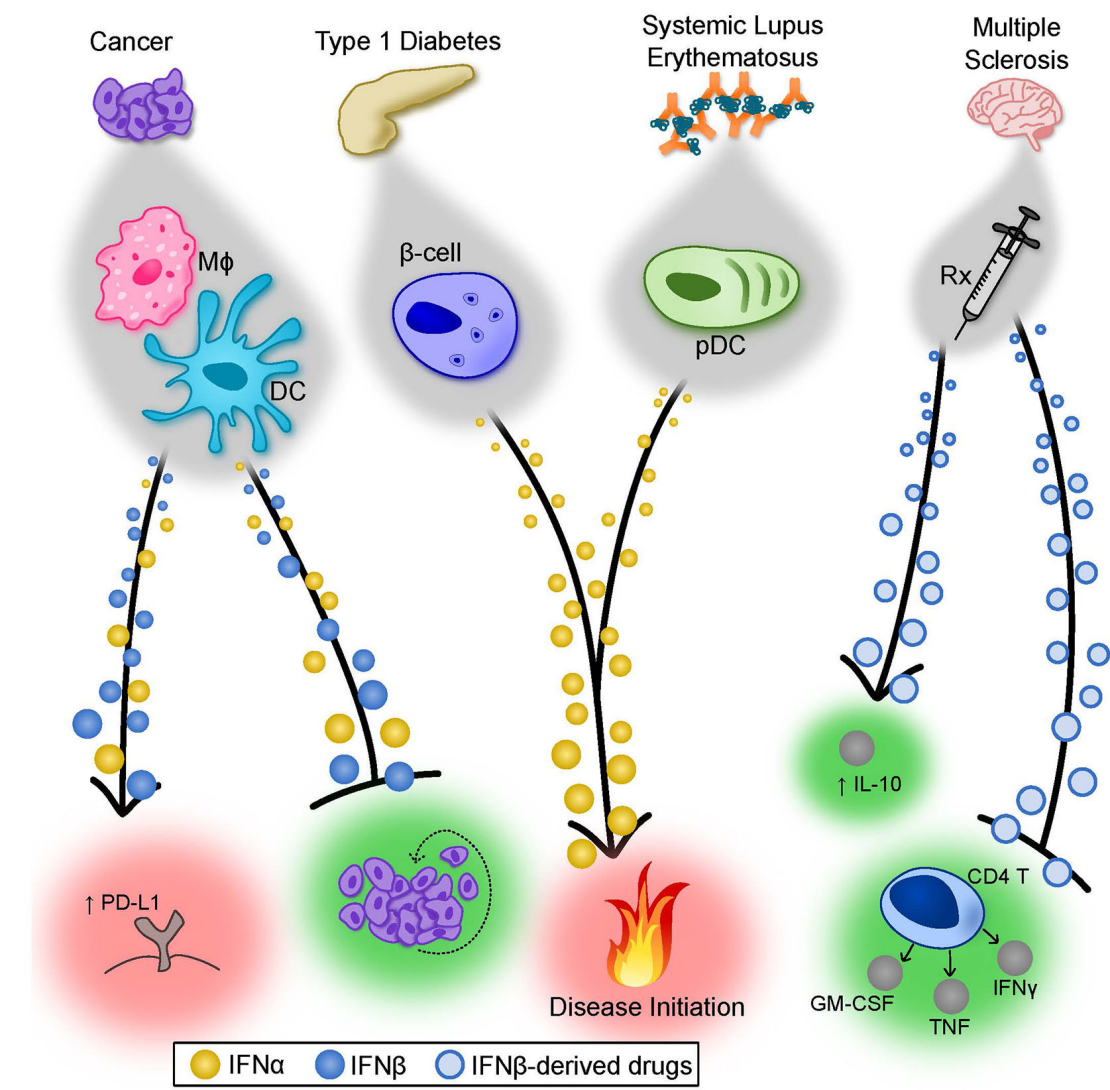
Summary of the Properties of IFNα and IFNβ in cancer and autoimmunity. Type I IFNs display both unique and overlapping properties in various disease states. In cancer, depending on the tumor and degree of metastases, both IFNα and IFNβ can contribute to tumor rejection by directly limiting tumor cell proliferation (depicted) but also through modulation of antitumor immune responses (not depicted). In certain cases, type I IFNs can induce PD-L1 expression on tumor cells, suppressing immune-mediated killing of the tumor. The factors that cause type I IFNs to exert detrimental effects remain poorly understood. In T1D, there is evidence that IFNα subtypes play an important role in pathogenesis. Forced expression of IFNα by pancreatic β-cells accelerated the onset and severity of T1D in a mouse model, and patients receiving IFNα therapy for treatment of other diseases have a higher incidence of T1D. Similarly, immune complex-driven activation of pDCs induces robust IFNα production, which may participate in initiation of SLE. Finally, IFNβ-derived therapeutics have well-established efficacy for treating MS patients. Though still largely debated, the mechanism of protection mediated by IFNβ is complex and possibly includes limiting cytokine production from pathogenic CD4^+^ T cells and augmenting IL-10 production in a number of cell types. β-cell, pancreatic β-cell; DC, dendritic cell; IL, interleukin; Mφ, macrophage; MS, multiple sclerosis; pDC, plasmacytoid dendritic cell; Rx, prescription drug; SLE, systemic lupus erythematosus; T1D, type I diabetes.

The majority of reports from animal models and the clinic demonstrate that type I IFNs play an important protective role in enhancing anti-tumor immune responses and restricting tumor growth [reviewed in (222, 223)]. However, similar to persistent viral infections, the functions of type I IFNs in cancer can change throughout disease course, and there is evidence that, in certain contexts, IFN might act as a barrier to efficacious checkpoint-blockade therapy [reviewed in (224)]. Below we discuss the actions of endogenous IFNα/β and IFN-based therapies in animal models and clinical studies (**Figure 2**).

### Animal Studies: Endogenous Type I IFN Activity

An early study showed that mice transplanted with human tumors and treated with neutralizing antibodies to type I IFNs demonstrated exacerbated tumor growth and metastasis compared to controls, suggesting a protective role for endogenous type I IFN activity (225). Since this finding, we now know that endogenous type I IFN can mediate tumor rejection through signaling on immune cells or tumor cells.

A seminal paper showed that type I IFN signaling on host hematopoietic cells was crucial for development of anti-tumor immune responses (226). Using conditional IFNAR1 deletion, bone marrow chimeras, and adoptive transfer experiments, a number of studies have shown that type I IFN signaling on several types of immune cells is important for immunity in cancer. For instance, type I IFN signaling on DCs, but not granulocytes or macrophages, was required for rejection of highly immunogenic tumors (227). Additionally, *Itgax-Cre^+^ Ifnar1* ^fl/fl^ (CD11c-Cre) mice showed diminished cross presentation by DCs to CD8^+^ T cells, which likely contributed to their failed tumor rejection (227, 228). In an NK cell sensitive tumor model, endogenous type I IFN was required for NK cell-mediated tumor rejection and homeostasis (229).

Other studies have shown that type I IFN signaling on tumor stromal cells may be important for controlling tumor burden. *In vivo*, both IFNα and IFNβ have antiangiogenic activity *via* signaling on vascular endothelial cells to downregulate growth factors such as vascular endothelial growth factor (230, 231). Stromal cells such as mesenchymal stem cells may play a role in controlling tumor growth by producing IFNα in order to enhance NK and CD8^+^ T cell responses (232). However, extended low level IFN signaling on tumor cells may render them resistant to apoptosis and immune-mediated killing (233, 234). These differences highlight the complexities of type I IFN actions and the need to delineate cell-type specific IFN signaling and consequent gene regulation.

Limited studies have directly compared the endogenous functions of individual IFNα/β subtypes in cancer models, but there have been a few studies conducted with IFNβ-deficient mice. *Ifnb* ^−/−^ mice showed expedited tumor growth, enhanced angiogenesis, and increased neutrophil infiltration to the tumor compared to WT mice (235–238). These findings demonstrate that endogenous IFNβ is important for the host anti-tumor response, but the specific signaling pathways downstream of IFNβ and cell types mediating these effects remain unclear. The direct contributions of endogenous IFNα remain uninvestigated, so much work is needed to fully characterize the contribution of endogenous IFN in tumor rejection.

### Animal Studies: Type I IFN-Based Therapies

The possibility that IFNs might be therapeutically useful in cancer was first shown in the early 1970s, when crude preparations of were administered to mice with syngeneic tumors increased their survival compared to untreated mice (239, 240). IFN therapies have been quite effective against hematological cancers, including hairy cell leukemia and chronic myelogenous leukemia but vary in efficacy against solid tumors, such as melanoma [reviewed in (222, 223, 241, 242)]. Below we discuss various therapeutic strategies derived from either IFNα or IFNβ subtypes. Collectively, these studies show that IFNα and IFNβ are able to promote a similar range of immunomodulatory and antitumor effects, so studies that directly compare the activities of IFNαs and IFNβ are needed to discern if there are bona fide differential properties.

#### IFNα-Based Therapies

Derivatives of IFNα2b have long been used in the clinic, but toxicity issues are associated with systemic administration and persistent use. Consequently, many groups have sought ways to increase IFNα expression with more precision. An influential study developed RNA-lipoplexes encoding neoantigens or endogenous self-antigens, which yielded rapid and robust IFNα production by macrophages and DCs (IFNβ induction was not determined) (243). Importantly, these RNA-lipoplex vaccines were able to mediate rejection of several different types of aggressive tumors in mice (243). Another group developed a method to genetically modify human hematopoietic stem cells (HSCs) to express HuIFNα2b, but only in differentiated monocytes (244). The engineered HSCs were able to repopulate immunodeficient mice and effectively inhibit tumor progression in a murine breast cancer model (244). AcTakines (Activity-on-Target), which are optimized cytokines that only act on cells for which they are targeted, represent another interesting alternative to traditional IFN therapies. Indeed, CD20-targeted IFNα2b-derived AcTaferon reduced lymphoma and melanoma tumors engineered to express CD20 (245, 246). Increasing tumor cell production of IFNα is another approach, and a very recent study demonstrated that IFNα subtypes are not all equal in their antitumor properties. B16 melanoma cells were engineered to overexpress IFNα2, α4, α5, α6, or α9, but only IFNα2- and α9-expressing tumors were effectively controlled in an adaptive-immunity dependent manner (247). Other studies have used a variety of genetic engineering methods to augment IFNα production in the tumor microenvironment and improve antitumor immunity (248–251).

#### IFNβ-Based Therapies

Derivatives of IFNα2 have been the focus of most IFN-based therapies, but several studies have explored the effect of IFNβ during various models of cancer. IFNβ treatment of transformed human mammary epithelial cells *in vitro* led to a less aggressive state (252). Another group showed that treating mice with an anti-tumor antibody fused to IFNβ increased clearance of antibody-resistant tumor cells by increasing cross presentation by tumor-infiltrating DCs and activation of CD8^+^ T cells (253). Unfortunately, this treatment also upregulated the inhibitory molecule PD-L1 on tumor cells, but this negative effect was overcome with co-administration of anti-PD-L1 antibody (253). Another group transduced induced pluripotent stem cell (iPSC)-derived myeloid cells with an IFNβ-encoding lentivirus to treat disseminated gastric cancer (254). When injected into immunocompromised mice, the modified myeloid cells accumulated in the tumors and inhibited growth of the peritoneally disseminated cancer (254). Lastly, intratumoral injection of an mRNA encoding a fusion protein consisting of IFNβ and the ectodomain of transforming growth factor-β receptor II enhanced DC activation of CD8^+^ T cells *in vitro* and promoted rejection of the TC-1 tumor cell line *in vivo *(255).

### Human Studies

The antitumor and immunomodulatory effects of IFNα therapy have been demonstrated in the treatment of a variety of cancers, and here we present a few representatives. IFNα-derived therapies are the only approved adjuvant therapies in melanoma patients after surgical resection, and immunomodulatory actions, such as increased tumor-infiltrating cells and decreased circulating T-regulatory cells, are key mechanisms of action [reviewed in reference (242)]. After being replaced with tyrosine kinase inhibitors like imatinib, interest in IFNα-based therapy has recently reemerged for treatment of chronic myeloid leukemia (CML) [reviewed in reference (241)]. This is because there is evidence that IFNα therapy is able to target and sensitize the rare CML stem cell population to subsequent killing by chemotherapy, whereas imatinib is more effective against more differentiated CML progenitors (256, 257). Lastly, an analysis of matched primary breast cancer tumors and bone metastases revealed that primary tumor cells expressed *IRF7*, whereas metastases consistently demonstrated downregulation of *IRF7* expression (258). This may suggest that IRF7-mediated IFNα production in primary tumors is an important factor for limiting metastases, but further studies are needed to determine if this is an IFNα-specific effect or if there is also a role for IFNβ. Fewer clinical studies have been conducted with IFNβ-derived therapies, but there is evidence that IFNβ also plays a protective role in tumor rejection. Increased IFNβ mRNA expression significantly correlated with improved survival in patients with triple-negative breast cancer, though the mechanism is undetermined (252). *In vitro* studies have shown that IFNβ is more potent in inducing apoptosis in melanoma cells compared to IFNα (259). The relevance of this differential potency has yet to be extensively explored *in vivo*.

### Detrimental Effects of Type I IFNs in Cancer

Despite all the evidence that type I IFNs can facilitate protective antitumor immune responses, IFNs can also impede cancer therapies. We provide just a few mechanistic examples. Persistent type II IFN signaling on tumors can result in PD-L1-dependent and PD-L1-independent resistance to immune checkpoint blockade, and the authors identified a role for type I IFNs in maintaining PD-L1-independent resistance (233). Radiation and chemotherapy stimulate immune-mediated destruction of tumor cells partly through induction of type I IFNs (260–264). However, recent work showed that conditional deletion of IFNAR1 on tumor cells enhanced responsiveness to radiation therapy through increased susceptibility to CD8^+^ T cell-mediated killing (265). Lastly, oncolytic viruses can preferentially kill cancer cells, but tumor responsiveness to type I IFN activity confers resistance to this therapeutic method. One study showed that IFNα and IFNβ differ in their ability to confer resistance to oncolytic virus treatment *in vitro*. Exogenous IFNβ more effectively prevented oncolysis of human head and neck squamous cell carcinoma cells by vesicular stomatitis virus compared to IFNα, but differential effects were not observed for normal keratinocytes or endothelial cells (266).

### Remarks on Cancer Studies

Collectively, this large body of cancer studies has shown that the roles of type I IFNs are complex and likely context specific. The extensive use of IFNα-derived therapies to treat a number of cancers in the clinic has greatly increased our understanding of the range of IFNα properties *in vivo*. Cancer models are uniquely advantageous for studying protective immunomodulatory effects of IFNs compared to infection models because pathogen load is not a confounding factor. Despite the large body of work suggesting the benefits of type I IFN signaling in cancer, the actions of specific IFN subtypes, for the most part, remain undefined. The beneficial effects of indirect activators of type I IFNs, such as the RNA-lipoplexes (discussed above) or STING agonists, may be due to their ability to induce multiple IFN subtypes with either overlapping or unique functions (222, 244). The heterogeneity of cancer makes it all the more important to appropriately stratify patients to ensure a beneficial effect of treatment.

## Autoimmunity

Type I IFNs have emerged as critical mediators of autoimmunity. Patients with a variety of autoimmune diseases display serum type I IFN signatures, and IFN treatments for other diseases have correlated with the development of autoimmunity. These observations have led to the assumption that type I IFNs may contribute to autoimmunity pathogenesis. However, IFNβ-derived therapeutics have been used to treat multiple sclerosis, highlighting that caution is warranted in attempting to summarize the mechanisms of autoimmune disorders. Below we outline the current understanding of the roles of IFNα and IFNβ during systemic lupus erythematosus, type 1 diabetes, and multiple sclerosis (**Figure 2**). This is not an exhaustive analysis of autoimmune disorders, and active research is exploring the function of type I IFNs in other disorders, such as rheumatoid arthritis and Sjögren’s syndrome (267, 268).

### Systemic Lupus Erythematosus

Systemic lupus erythematosus (SLE) is an autoimmune disease that affects organs such as the skin, joints, kidneys, and CNS (269). A type I IFN gene signature in the blood of SLE patients is well established (270–272). Additionally, a number of genetic risk factors for SLE are associated with type I IFN production or signaling, including *IRF5, IRF7, IRAK1*, and *TYK2* [reviewed in reference (273)]. The majority of patients (70–80%) develop anti-nuclear autoantibodies (ANA), which form immune complexes with extracellular nucleic acids and induce production of type I IFN, especially IFNα, by pDCs (274). Type I IFNs promote disease by signaling on a variety of immune cells, including DCs, B cells, and T cells (275–277). It has been shown that IFNα or IFNβ treatment *in vitro* induced different transcriptional programs in DCs, with IFNα-primed DCs demonstrating increased phagocytic uptake of apoptotic cells and nucleic acids (278). Given the prevalence of IFNα in the serum of SLE patients and role of pathogenic responses to nucleic acids, the impact of IFNα versus IFNβ on DC activation in the context of SLE might be an interesting topic to interrogate.

A recent study from Klarquist *et al.* sought to dissect the effect of type I IFN signaling on CD4^+^ T cells and B cells on the development of T follicular helper cells, germinal center B cells, and plasmablasts. They found that IFN signaling decreased the threshold for B cell receptor signaling, increased MHC-II expression, and promoted germinal center B cell function, thus lowering the threshold for autoreactive B cell activation (276). They also found that type I IFN protected T follicular helper cells from NK cell-mediated death, thus further promoting B cell responses (276). Other studies suggest that IFNα may further drive SLE by increasing production of multiple TNF family members, such as BAFF and APRIL, which promote B cell survival and can drive SLE pathogenesis (279–281). Due to the apparent pathogenic role of IFNα during SLE, attempts have been made to neutralize type I IFNs in SLE patients (282–287). Both anti-IFNα and, more recently, anti-IFNAR1 therapies have been tested (282–287). Both treatment strategies showed disparate efficacy in patients, so further work is needed to clarify if this type of therapeutic intervention would be beneficial for patients. It might be that IFNα only plays a key role in the initiation and early stages of disease, so the disease stage may be important in stratifying patients [reviewed in reference (288)].

### Type 1 Diabetes

Type 1 diabetes (T1D) is a chronic, autoimmune disease caused by the immune-mediated destruction of pancreatic β-cells that leads to insulin deficiency and hyperglycemia (289). A blood type I IFN signature in T1D patients precedes the development of autoantibodies and disease (290–293). One study detected a significant increase in expression of IFNα subtypes, but not IFNβ, in postmortem pancreas specimens from T1D patients compared to control subjects (290). Moreover, many genetic polymorphisms associated with T1D are involved in the type I IFN response such as *MDA5* and *TYK2 (* 294–296). Altogether, these findings suggest a detrimental role for type I IFNs in T1D. A role for type I IFNs in the development of T1D is supported in animal models. An early study showed that forced constitutive IFNα expression by pancreatic β-cells in mice resulted in hypoinsulinemic diabetes and pancreatic inflammation (297). Additionally, non-obese diabetic (NOD) mice, a common model for T1D, showed elevated IFN-inducible transcripts in the pancreatic islets prior to disease onset, and treatment of young NOD mice with anti-IFNAR1 mAb delayed the onset and decreased the occurrence of T1D (298, 299). Collectively, these findings suggest that type I IFN signaling, especially in the pancreas, may play a key role in initiating T1D.

LCMV can be employed as a viral model of T1D, in which mice transgenically express LCMV glycoprotein (GP) under the control of the rat insulin promoter (*Rip*-LCMV) (300). Development of *Rip*-LCMV T1D is dependent on type I IFN (301, 302). Recent work showed that anti-IFNAR1 mAb treatment reduced blood glucose to normal levels and prevented destruction of pancreatic islets (302). Importantly, they also showed that pan-IFNα (α1, α4, α5, α11, and α13) mAb blockade, but not IFNβ blockade, was able to recapitulate the anti-IFNAR1 phenotype, demonstrating a distinct role for IFNα subtypes in promoting pathogenesis in the *Rip*-LCMV T1D model. A similar detrimental role for IFNα is suggested in human disease. IFNα therapy for HCV in individuals genetically predisposed to T1D induced or exacerbated the development of T1D (303). Moreover, a recent study showed that a subset of *AIRE*-deficient patients who developed autoantibodies specific for IFNα, especially IFN-α1/13, IFN-α5, and IFN-α14, were less likely to develop T1D, whereas patients who failed to generate these antibodies developed T1D (304). Altogether, animal and human studies suggest a detrimental role of type I IFNs in T1D, and IFNα subtypes appear to play a dominant role in disease development and pathogenesis.

### Multiple Sclerosis

Multiple sclerosis (MS) is a chronic, autoimmune disease of the CNS in which immune cells target and destroy the myelin sheath surrounding neurons, leading to neurodegeneration (305). Similar to other autoimmune conditions, MS patients can show a serum type I IFN signature, but this signature is relatively low when compared to SLE patients (306, 307). However, in strong contrast to SLE and T1D, type I IFNs, do not appear to play a detrimental role. In fact, IFNβ was the first FDA-approved therapy for MS (308–311). However, due to its flu-like side effects and the availability of more effective treatments, it is no longer the preferred therapy for MS patients (312). Even though IFNβ treatment is currently less preferred in clinical use, animal models and clinical studies (discussed below) have revealed important insight into the properties of IFNβ *in vivo*.

#### In Vitro and Animal Studies

Experimental autoimmune encephalomyelitis (EAE), a mouse model of MS, has provided mechanistic insight into the protective actions of IFNβ (313). Mice lacking IFNβ, IFNAR1, or IRF7 showed exacerbated clinical EAE compared to WT mice, perhaps due to greater T cell infiltration and increased proinflammatory cytokine production in the CNS (314–316). Unexpectedly, mice that lack IRF3 showed significantly lessened clinical disease compared to WT mice, and this seemed to be due to a cell-intrinsic defect in the development of T helper type 17 (T_H_17) cells (317). Indeed, T_H_17 versus T_H_1 skewing can drastically influence the impact of IFNβ treatment in EAE. IFNβ treatment was effective in reducing EAE severity in T_H_1-induced EAE but worsened disease in T_H_17-induced EAE (318). Thus, depending on the skewing of the T helper responses and method of induction of EAE, IFNβ may be protective or pathogenic.

Many cell types respond to IFNβ therapy in EAE. Deletion of *Ifnar1* on myeloid cells including macrophages, monocytes, granulocytes, and microglia, but not neuroectodermal cells, resulted in increased severity of EAE symptoms, suggesting that IFNβ mediates its protective effects, in part, by acting on these cells (315). Mice treated with TLR3 or TLR7 agonists display reduced disease severity associated with increased type I IFN production by pDCs and other antigen presenting cells (319, 320). Other reports have also suggested that IFNβ signaling on T cells curbs their pathogenicity (321, 322). Furthermore, type I IFN signaling on conventional DCs limited their migration to the CNS and prevented their activation of T_H_17 cells during EAE (323, 324). The tissue resident antigen presenting cells in the CNS, microglia, may also play a role in the type I IFN response during EAE. Type I IFN signaling on microglia promoted clearance of myelin debris by increasing their phagocytic activity (325, 326). Finally, a study identified a role for type I IFN signaling on astrocytes to suppress CNS inflammation during EAE (327).

Clearly IFNβ is able to induce protective effects during EAE, and a recent report demonstrated that sustained low-dose IFNα1 delivery *via* an adeno-associated viral system prevented the onset of disease in EAE (328). This therapeutic effect was associated with regulatory T cell expansion, and myelin-specific effector T cells displayed reduced proliferative capacity, decreased proinflammatory cytokine production, and increased expression of IL-10 and PD-1 (programmed cell death protein 1) (328). Another study showed that a systemic high dose of MuIFNα11 was able to initially delay EAE in mice but ultimately caused significant toxicity and mortality; however, when IFNα activity was targeted to DCs (Clec9A-targeted AcTaferon), they found efficient protection from EAE (329). These findings suggest that IFNβ might not be unique in its ability to confer protection in EAE, but more work is needed to determine what factors cause IFNα treatments to yield detrimental effects or protective effects.

#### Human Studies

IFNβ was the first FDA-approved therapy for MS (308–311). However, due to its flu-like side effects and the availability of more effective treatments, it is no longer the preferred therapy for MS patients (312). Observations from patients suggest that IFNβ therapy likely acts through multiple mechanisms, such as influencing immune cell recruitment and activation. First, IFNβ treatment correlated with decreased new brain lesions and increased soluble VCAM-1 in patient serum, suggesting that modulating immune cell entry to the CNS is one potential mechanism of IFNβ therapy (330). In addition to impacting cell recruitment, IFNβ treatment may also regulate survival of immune cells since an increase in proapoptotic genes was observed in peripheral immune cells isolated from IFNβ-treated patients (331, 332).

Pathogenic T_H_1 and/or T_H_17 cells likely play an important role in MS, and IFNβ therapy may limit the proliferation of pathogenic T cells and modulate their cytokine production (332, 333). IFNβ therapy is likely more effective in individuals with a T_H_1 driven disease, since high serum IL-17F levels correlated with a poor response to IFNβ therapy (318). A number of cell types are likely involved in protective IFNβ treatment. For example, IFNβ treatment of MS patients can induce IL-10 production by myeloid cells, but treatment can also suppress production of granulocyte-macrophage colony-stimulating factor (GM-CSF), IFNγ, and TNF by effector T cells (334–340). Additionally, in patients that responded to IFNβ therapy, treatment induced T regulatory cells that produced IL-10 and expressed PD-L1 (341, 342). Altogether, the protective mechanisms that underlie IFNβ therapeutic effects likely involve direct or indirect actions on effecter T cells. A better understanding of these mechanisms would likely reveal important information about the functional capacity of IFNβ *in vivo*.

### Remarks on Autoimmune Studies

A large proportion of patients with SLE or T1D show a type I IFN signature in their blood, and many studies have shown that type I IFNs promote pathogenesis in these autoimmune disorders. There is strong evidence implicating the IFNα subtypes in initiation and progression of SLE and T1D, but at this time, a role for IFNβ cannot be entirely ruled out—direct functional comparisons of IFNα versus IFNβ would be needed to draw that conclusion. Altogether, the specific pathogenic functions of type I IFNs during autoimmune disorders are likely tissue specific. A recent study performed gene-expression profiling of structural cells from 12 different tissues and found that the responses of the cells to stimuli were tissue-specific, thus identifying the stroma as an important regulator of tissue-specific immune responses (343). While there is clear evidence that type I IFNs can modulate pathogenic autoimmune responses, it is important to know how systemic IFNα activity might promote cell-type specific effects in diseased versus nondiseased tissues in disorders like T1D that target a particular tissue, but also in diseases like SLE that have multi-organ effects.

In contrast, blood from MS patients do not display as robust a type I IFN signature as SLE or T1D patients, and many studies have demonstrated that IFNβ treatment has therapeutic properties in animal models of MS and in affected individuals. The protective functions of IFNβ are complex and likely include modulating immune cell recruitment and activation directly through action on immune cells and indirectly through action on brain resident cells. The functions of IFNαs in MS are less clear. There might be conditions, such as very low doses or when targeted to a specific cell type, in which IFNα subtypes are also protective. Careful comparison of IFNβ versus IFNα dose responses in EAE might uncover novel mechanisms for differential functions among type I IFNs *in vivo*.

## Concluding Remarks

Whether type I IFNs have a net beneficial or detrimental effect on disease outcome depends on a variety of factors including the timing and magnitude of induction relative to disease onset, the duration of expression, the specific subtypes induced, the cell types responding, and likely other factors. Progress is needed in understanding the spatiotemporal induction of the various type I IFN subtypes *in vivo*, as well as the cell types responsible for type I IFN production. A lack of tools to differentiate between different subtypes has hindered progress in this area. Quantitative reverse transcription polymerase chain reaction has been a useful technique for quantifying specific IFN subtypes, and single-molecule array (Simoa) digital ELISA technology was demonstrated to detect IFN in blood with high sensitivity (344). However, there is a need for licensed antibodies against individual subtypes that are able to neutralize in animal models and reliably stain tissue sections to more accurately determine the timing of expression at the tissue level.

Transcriptomic approaches have successfully differentiated type I and type III ISG signatures in organoid cultures (345). Because the effects of type I IFN are pleiotropic, there is a need to delineate the ISGs responsible for the protective and pathogenic functions of type I IFN subtypes in a given context and to understand how cell-type specificity might affect expression of those genes. A recent report profiled gene-expression networks of fibroblasts, endothelial, and epithelial cells isolated from multiple tissues and revealed tissue-specific signaling networks (343). A similar approach or spatial transcriptomics, which yields gene expression profiles in intact tissue sections, would be powerful tools to unravel the cell type-specific responses to different type I IFN subtypes *in vivo* (346).

Lastly, given that many type I IFN subtypes have expanded independently after mammalian speciation, there is a great need for tools to allow the study of human type I IFN subtypes in animal models. Immune-humanized mice and hybrid IFNAR (HyBNAR) mice, which transgenically encode variants of IFNAR1/2 that contain the human extracellular domains fused to the transmembrane and cytoplasmic segments of murine IFNAR, have both been used to study HuIFN in mice (347). These two systems are helpful in contexts where immune cells are the predominant sources of and responders to type I IFN or in studies administering exogenous HuIFN, but they do not permit loss-of-function studies, exclude the impact of endogenous IFN expression by stromal cells, and IFNAR1/2 transgenes are likely more highly expressed than endogenous IFNAR1/2. Overall, a concerted effort to address this lack of tools will go a long way toward increasing our ability to directly compare the expression and functions of distinct type I IFN subtypes, which will undoubtedly generate new strategies to augment or dampen the type I IFN pathway for biomedical purposes.

## Author Contributions

LEF and MCL conceptualized and drafted the manuscript, as well as created the figures and tables. LEF, MCL, and DJL all reviewed and edited the manuscript. All authors contributed to the article and approved the submitted version.

## Funding

LEF was supported by a National Institutes of Health (NIH) postdoctoral research training grant (T32 CA009547) (https://www.nih.gov/). MCL was supported by a predoctoral research training grant (T32 AI007163) and by an F31 fellowship (AI149999-01A1) (Ruth L. Kirschstein Predoctoral Individual National Research Service Award) from the NIH (https://www.nih.gov/). DJL was supported by the NIH (R01 AI127513 and R21 AI135490) (https://www.nih.gov/).

## Conflict of Interest

The authors declare that the research was conducted in the absence of any commercial or financial relationships that could be construed as a potential conflict of interest
